# Investigation of the Lower Resistance Meridian: Speculation on the Pathophysiological Functions of Acupuncture Meridians

**DOI:** 10.1155/2014/107571

**Published:** 2014-12-02

**Authors:** Weisheng Yang

**Affiliations:** ^1^Department of Physics, Peking University, Beijing 100871, China; ^2^Institute of Physics, Chinese Academy of Sciences, Beijing 100080, China

## Abstract

It was pointed out in the two earlier papers of the present author that the meridians are in fact zones in the loose connective tissue containing richer interstitial fluid and thus are lower-resistance passages for diffusion of meridian-signal carriers or mediators. Moreover, a hypothesis, which incorporates the wide variety of functions of the loose connective tissue, the circulatory system, and the nervous system into the meridian function, has been proposed and in the hypothesis the mast cell plays some key roles. In the present paper, considering also the latest knowledge on cell migration along with some existing experimental results, it is further pointed out that meridians ought to be lower-resistance passages for chemotactic migration of cells and mast cells can indeed migrate longitudinally along meridians. Finally, the present paper points out that if we add the last two points to the hypothesis and keep in mind that mast cells have been known very recently to be versatile regulators of inflammation, tissue remodeling, host defense, and homeostasis, the rich pathophysiological functions of the meridian pointed out by the traditional Chinese medicine can be understood quite naturally.

## 1. Introduction 

Cell migration means movement of cells after receiving a signal for that or encountering a gradient of some substances. In the course of movement a cell is polarized into an approximately oblong shape firstly and then repeats the cyclical process in which the cell extends protrusions at its front and retracts its trailing end. For a cell to advance the newly extended protrusions must attach stably through integrins to the surroundings, providing a means of traction; meanwhile, the existing adhesions at the cell rear must disassemble promptly. The actin cytoskeleton and the myosin II interacting with it, along with the extracellular matrix, are the material basis of cell migration. Besides, many other substances are also involved in the subtle control of cell migration [1–3].

Cell migration is involved in many important physiological processes, such as embryonic morphogenesis, wound healing (tissue repair and regeneration), bacterial infection, and immune responses, and it also drives disease progression such as in atherosclerosis, mental retardation, chronic arthritis, asthma, cancer genesis, and metastasis. Our liaison with cell migration begins shortly after conception, accompanies us throughout life, and often contributes to our death. Mechanism and regulation of cell migration are of current interest in cell biology. The last two decades have witnessed enormous advances in our understanding of cell migration [2, 3].

It has been suggested in the two earlier papers of the present author that meridians are zones in the loose connective tissue containing relatively richer interstitial fluid (tissue fluid) [4] and that meridians are passages with lower resistance for diffusion of meridian signal carriers, such as histamine and other mediators [5]. Considering the great importance of cell migration and based on the newest knowledge of it, especially the very recent progresses in cell migration in the three-dimensional (3D) matrix [6, 7], the present paper explores the possible relations between the meridian function and cell migration.

## 2. Meridians Are Lower-Resistance Passages for Cell Migration in Extracellular Matrix

Cell migration has been studied extensively in 2D cell culture models. However, discrepancies between the behavior of cells in culture and in 3D extracellular matrix (ECM) in vivo are significant and, moreover, the required techniques are more complicated for in vivo studies; for instance, real-time in vivo imaging technologies are necessary [8]. Despite of that, systematic and in depth knowledge on mechanisms and regulation of cell migration in 3D ECM has been acquired in recent years [6, 7].

Cell migration involves different cell types and tissue environments. The size, shape, and the ability of deformation are quite different for different types of cells; meanwhile, the composition and structure of the three major types of 3D ECM where cells often migrate, that is, the dense connective tissue, loose connective tissue, and tightly packed basement membrane, are also very different [7], and mechanism of cell migration varies dramatically. However, prespecified cell-type-specific patterns of cell migration can be classified into single cell migration and collective migration modes, and the former is further classified into amoeboid and mesenchymal, whereas the latter is further classified into cell sheets, strands, tubes, and clusters. The intrinsic molecular programs of these migration types are associated with a characteristic structure of the actin cytoskeleton, as well as the cell-type-specific use of integrins, matrix-degrading enzymes, cell-cell adhesion molecules, and signaling towards the cytoskeleton [6]. As a result, the mesenchymal migration of single cells is much slower than the amoeboid migration, as one can see from Table 1. This is because, to form proteolytic matrix defects already has to take some time; moreover, the force required for pulling a rigid cell through the generated narrow defects could not be very small, thus making formation and turnover of the focal contacts required for giving rise the force also take quite some time. As for the collective migration, cell sheets, strands, tubes, and clusters are much larger and rigid, thus making the required amount of path generation larger and the strength of the required adhesions stronger than those in single cell mesenchymal migration and, in turn, making their migration slower [6]. Note that the classifications of cell migration are not absolute, meaning that if the relevant condition or parameter varies, transition between different migration modes may happen, such as mesenchymal-amoeboid transition [6, 9].

Now, we turn to the cell migration in meridians. In this regard, it has been pointed out in the literature that whatever the cell type and/or migration mode is, the matrix resistance of the loose connective tissue is always lower than that of the densely packed connective tissue, because the former is consisted mainly of irregularly orientated collagen fibers and elastic fibers with large spaces in between, whereas the latter is consisted of some immobile fibroblasts and densely packed collagen fibers [7]. Besides, we have recalled above that meridians are zones in the loose connective tissue containing relatively richer interstitial fluid (tissue fluid) [4]; in other words, meridians contain more spaces in comparison even with other places in the loose connective tissue and thus give rise to less resistance to cell migration. As a result, (i) for cells usually making amoeboid migration, migrating in meridians encounters the least resistance and thus can have the highest speed; (ii) for cells usually migrating in the mesenchymal mode, moving in meridians meets lower resistances and thus makes the mesenchymal-amoeboid transition possible [9] and in turn can have the highest speed; (iii) for cells or cell groups that could make only mesenchymal migration, the path generating task in meridians also becomes less tedious and thus their speed can also be high in meridians. Consequently, meridians must be lower-resistance (or, fast-speed) passages for single cells as well as cell groups migrating in ECM.

## 3. Meridians Are Lower-Resistance Passages for Cell Chemotactic Migration in Extracellular Matrix 

It should be pointed out that to become a passage for cell chemotactic migration only to be able to give rise to a lower resistance to cell migration is not enough, because cell chemotactic migration, by definition, is a sort of migration driven by some chemoattractant. In other words, a lower-resistance passage for cell chemoattractant migration must be also a lower-resistance passage for diffusion of chemoattractants. For meridians this is quite obvious, because there is the effective cross section for diffusion of chemoattractants, that is, the relative area not occupied by any kind of cells and fibers but by interstitial fluid is larger compared to that in nonmeridian places [4, 5]. Therefore, meridians are lower-resistance passages for diffusion of chemoattractants as well as for cell migration and thus are fast-speed passages for cell chemotactic migration in ECM.

Actually, one can find similar thoughts from the literature: “Besides leukocyte shape change and contact guidance, additional (patho)physiological mechanisms are likely to support non-proteolytic lymphocyte trafficking through 3D tissues. In vivo, regions of loose fibrillar collagen networks support oedematous swelling and reversible widening of matrix gaps in response to vasodilatation and inflammation. Local edema is likely to generate biomechanically suitable pathways for rapid leukocyte trafficking independent of proteolytic matrix degradation. In the dermis, such loose connective tissue zones, which are closely mimicked by collagen matrices, include perivascular spaces located along blood vessels, adjacent to basement membranes, and in the dermal papillae. These “trafficking highways” are preferentially used by passenger leukocytes upon acute and chronic inflammation, such as in eczema [10].” It is not difficult to see that these thoughts are quite similar with our suggestions that meridians exist in the human body (i.e., zones in the loose connective tissue containing relatively richer interstitial fluid), that meridians are high-speed passages for cell chemotactic migration, and that the activated mast cells in meridians can release a variety of mediators like histamine, which can enhance the local microcirculation and in turn induce local edema. The only difference is that in the literature the concept of the meridian was not introduced.

However, it should be pointed out that, on one hand, in the traditional Chinese medicine the key issue is the so-called along-meridian transmission or connection [11], and the 12 major meridians are aligned mainly longitudinally, whereas on the other hand, so far only cell migrations like extravasation of cells from blood vessels to tissues [12, 13], that is, only lateral cell migrations, have been studied.

A natural question then is what can be the major function of these longitudinal lower-resistance passages of cell migration in the human body. The answer can be found from the immune system, because (1) leukocytes always migrate in the amoeboid mode as single cells and thus can be more than 100 times faster than other types of cells [14]. (2) The life span of leukocytes is very long and especially lymphocytes may have a life span of years [15]. Therefore, it is meaningful for such cells to spend some time to migrate along meridians to some other places to play their roles. (3) Moreover, taking the route of “ECM-lymphatic system-circulatory system-ECM” would be very likely not only longer but also slower, because along this route penetrating through basement membranes by proteolytic degradation of extracellular matrix is unavoidable, whereas it is very time consuming [10].

However, limited by technical difficulties, investigations on cell migration had been carried out essentially with the 2D culture models [7]. Now, people have started to study border cell migration in vivo by using time-lapse live-cell imaging [8]; one would then expect to see such techniques applied in real time observations of cell migration along meridians in vivo and in the near future.

## 4. Mast Cells May Migrate Longitudinally along Meridians

Despite no real time observations of cell migration, especially long distance migration along meridians in vivo has been reported so far, on the basis of some published experimental results, we argue below that mast cells can indeed make long distance migration along meridians.

An earlier observation [16] of mast cells in subcutaneous connective tissue (fascia) in albino rats found that “In the adult rats, the mast cells in fascia are distributed not uniformly, they are oval in shape and are usually arranged in rows along the cell length with polarity. Many rows of mast cells are packed to form an elongated zone. Several zones oriented in the direction of the longitudinal axis of lower extremities are found.” Interestingly, the same observation also found that “in new born rats, mast cells are distributed uniformly and most of them are round in shape. The mast cells are arranged in a random way without polarity. In the arrangement of mast cells neither row nor zone is found, and there is no difference in distribution of them everywhere.” Unfortunately, no interpretations on these results were given by the authors [16]. Over the past few years, immense progress has been made in understanding cell migration. On the basis of that, our current understandings on the results of that old observation are the following.

Firstly, it has been known that during chemotactic migration cells are polarized, making their shape approximately oval with their long axis lying in the direction of migration, and that although spontaneous polarization may appear when there is no chemoattractant, in such cases the polarization direction is random and varying and thus does not cause migration in any fixed direction [6, 8]. Accordingly, the fact that in the adult rats the mast cells are “oval in shape and are usually arranged in rows along the cell length with polarity” indicates that the mast cells are often in chemotactic migration along the longitudinal direction of lower extremities. Actually, the fact that mast cells have been known recently as versatile regulators of inflammation, tissue remodeling, host defense, and homeostasis and that there are already a great deal of mediators or molecules having been identified as mast cell chemoattractants (see Section 5.1.2) is not surprising. Based on the same consideration, the results of that old observation also indicate that in the new born rats the mast cells are mostly quiescent or nonmigrating, very likely because they had never been invaded by viruses or pathogens till the observation was done.

Secondly, the fact that in the adult rats mast cells are usually arranged in rows along the cell length with polarity, that many rows are packed to form an elongated zone, and that several zones are oriented in the direction of the longitudinal axis indicates that for the adult rats, at least by the time of the observation, there exist already several “trafficking highways” in the fascia of their lower extremities, suitable for longitudinal migration for mast cells. In contrast, such “trafficking highways” do not exist in new born rats, indicating that rats are not born with such “trafficking highways.” Actually, this is not surprising either, because it has been known that tube-like proteolytic matrix defects may be left behind cells migrating in mesenchymal mode, and such newly formed matrix defects facilitate the migration of following cells along these paths of the least resistance ultimately leading to the migration of aligned cell strands following each other [17]. As for the “path generating pioneers” of the “trafficking highways” inside the adult rats, although they do not necessarily have to be but very likely were mast cells, because mature mast cells are not only permanent residents in the loose connective tissue, but also are known to have long life spans and versatile functions.

Now, knowing that meridians are lower-resistance passages for cell chemotactic migration and that mast cells may migrate longitudinally along meridians, what kind of extensions have to be or may be added to the hypothesis and what incorporates the wide variety of functions of loose connective tissue, circulatory system, and nervous system into the meridian function have been proposed in [5].

## 5. Extension to the Meridian Function Hypothesis Proposed Previously 

To facilitate the discussions bellow, we recall briefly the latest progresses in investigations of the mast cell function.

### 5.1. Summary of Mast Cell Functions

#### 5.1.1. Effector and Immunomodulatory Functions of Mast Cells

The possible roles of mast cells in heath and disease have been a topic of interest for over 125 years, especially in the last two decades. Now mast cells have been known as “versatile regulators” [18] of innate immune responses [19], adaptive immune responses [20], autoimmune responses [21], allergic diseases [22], effectors of immune responses [23], peripheral tolerance [24], host defense [25], tissue remodeling [26], and homeostasis [27].

More importantly, mast cells have recently been found to have, in addition to their positive immunomodulatory functions (such as promoting migration, maturation, differentiation, and function of immune cells), also negative immunomodulatory functions (such as suppressing sensitization for contact hypersensitivity, suppressing cytokine production by T cells and monocytes, and suppressing production of proinflammatory cytokines and chemokines by keratinocytes [28]).

The reasons that mast cells can have such rich immunomodulatory functions are that (1) there are many mediators that may serve as chemotactic factors for mast cells; (2) there are many mechanisms by which mast cells can be activated; (3) activated mast cells can release and produce a great variety of mediators and the mediators can induce different kinds of physiological functions.

#### 5.1.2. Mediators as Chemotactic Factors for Mast Cells

Many mediators may serve as chemotactic factors for mast cells, for instance, (1) chemokine (such as CCL5 (RANTES) [29], CXCL10 [30], and CXCL12 (SDF-1*α*) [31]); (2) cytokine (such as stem cell factor SCF [32, 33], transforming growth factor TGF-*β* [32, 33], tumor necrosis factor TNF-*α* [34, 35], interleukin IL-4 [34], IL-8 [34], and IL-15 [36]); (3) growth factors (such as basic fibroblast growth factor bFGF [37], vascular endothelial cell growth factor VEGF [37], platelet-derived endothelia l cell growth factor PD-ECGF [37], platelet-derived growth factor-AB PDGF-AB [37], and 5-hydroxytryptamine 5-HT [38]); (4) lipid mediators (such as prostaglandin PGE_2_ [39] and platelet-activating factor PAF [40]); and (5) other factors (such as products of complement activation (anaphylatoxins) C3a [41], and C5a [41]). Besides, a study has shown that histamine can also cause mouse mast cells chemotaxis toward histamine, but without affecting mast cell degranulation [42].

Such chemotactic factors, which are closely related to health and disease status of the body, may recruit mast cells to where they are needed, and then they may be activated by many different mechanisms.

#### 5.1.3. Mechanisms by Which Mast Cells Can Be Activated

There are also many mechanisms that may activate mast cells, for example [18], (1) via immune receptors (such as IgE, IgG_1_, and Ig-binding superantigens); (2) products of complement activation (such as C3a, C5a, C3b, and C4b); (3) ligands of toll-like receptors (such as peptidoglycan, viral RNA, lipopolysaccharides LPS, and flagellin); (4) bacteria and their products (such as* E. coli* FimH and virulence factors/toxins); (5) viruses (such as respiratory syncytial virus, influenza virus, and dengue virus); (6) parasites (such as* Schistosoma mansoni* and* Leishmania major*); (7) cytokines and inflammatory mediators (such as tryptase, SCF, IL-1, and IL-12); (8) endogenous peptides (such as nerve growth factor, substance P, CGRP and other neuropeptides, neurotensin, and antimicrobial peptides); (9) venoms or venom components (such as Sarafotoxin 6b, phospholipase A_2_, and mast cell degranulating peptide); (10) physical stimuli (such as UV light, cold, heat, pressure, and vibration). It is worthwhile to point that the stimuli applied in different acupuncture therapies are all physical stimuli being able to active mast cells.

After being activated by one of these mechanisms, which are closely relevant to health and disease status of the body as well as to the environment, mast cells can release their performed and/or de novo generated products (mediators).

#### 5.1.4. Major Mast-Cell-Derived Mediators

The mediators and their physiological effects (see Table 2) make mast cells able to regulate many functions of many different systems, including (1) epithelial functions (secretion and epithelial permeability); (2) smooth-muscle functions (peristalsis and bronchoconstriction); (3) endothelial functions (blood flow, coagulation, and vascular permeability); (4) immune functions (recruitment and activation of neutrophils, eosinophils, basophils, lymphocytes, monocytes, and macrophages, as well as migration and activation of dendritic cells); (5) host defense functions (degradation of endogenous toxic mediators, degradation of snake venom components, phagocytosis, and/or antimicrobial activity); (6) neuronal functions (neuroimmune interactions, peristalsis, and pain); (7) other tissue functions (wound healing and fibrosis).

Knowing all these, one would believe that mast cells are indeed “versatile regulators” of health status of the body.

### 5.2. Extension to the Meridian Function Hypothesis Proposed Previously

Based on the abovemetioned, we believe that, by adding the statements of “meridians are lower-resistance passages for cell chemotactic migration” and “mast cells can migrate longitudinally along meridians” to the hypothesis for the meridian function [5], which incorporates the wide variety of functions of the loose connective tissue, the circulatory system, and the nervous system into the meridian function, and meanwhile considering that mast cells have been known recently to be versatile regulators of inflammation, tissue remodeling, host defense, and homeostasis, or mast cells are “versatile regulators of health status of the body” [18], one may now start to think of or even to study the real scientific meaning behind the core of the meridian doctrine in the traditional Chinese medicine, that is, the so-called “*circulating “air and blood”,*” “*transferring responses,*” “*reflecting symptoms,*” and “*resisting diseases.*” The major difference between the hypothesis and the core of the meridian doctrine is that the former is expressed with the concepts and terminologies in modern medical physiology and pathophysiology. Moreover, if we also consider that mast cells can be activated by a variety of physical stimuli [18], such as UV light, cold, heat, pressure, and vibration, then the hypothesis may also provide us with some thoughts or hints in our evidence-based studies of the meridian function and/or the meridian therapy, like acupuncture, as some people have already started [43, 44].

Obviously, some of the latest knowledge on cell migration and mast cell functions needs to be verified by further investigations. Then, what are expected to be the key issues for future investigation of meridian functions, and what are expected to be the hardest issues?

## 6. Directions and Difficulties of Future Meridian Investigation 

Cell migration and mast cell functions are two hot topics for research in modern medical physiology and also are two difficult problems. No doubt, before the two are solved or basically so, one should not expect the concept of meridian to be accepted by physiology, especially the core of the concept, that is, the along-meridian longitudinal connections. Therefore, both cell migration and mast cell functions ought to be focused on in future meridian research. Then what are the difficulties?

The major difficulty in investigation of mast cell functions is the complexity of the problem itself. For instance, it has been noticed that [28] (1) for different mouse models of CHS (contact hypersensitivity) but under the similar conditions only changes in some small details may turnover an immunomodulatory function of mast cells from positive to negative or vice versa; (2) how individual positive or negative immunomodulatory functions can be induced or suppressed is relevant to variation in mast-cell population; and (3) in immune responses mast cells may first promote the sensitization phase of the response and could then help to initiate the local inflammation that occurs when the host is subsequently exposed to specific antigen and finally help to limit the extent of and/or resolve the ensuing inflammation and associated tissue pathology. Actually, considering the richness of mast cell chemoattractants, mast cell activation mechanisms, and mast cell-derived mediators along with their physiological effects, one would naturally expect to see these complexities.

The other difficulty in investigation of mast cell functions is that, in order to clarify the precise role that mast cells play in a complex process, it would be much easier if there could be samples with complete deficiency of mast cells as controls. Although for human this is impossible, for mouse this is basically possible. That is why in many experimental investigations on mast cell functions mice were used as samples [18–28]. Mouse and human are both mammalian and thus share many characteristics in common. This is why all kinds of mouse models are used in basic researches in medicine [45] or even in behavioral science [46]. However, mouse and human are nevertheless different, and thus many results obtained from mouse models still have to be tested in the future with human tests [45], but new difficulties are expected to emerge by that time.

The major difficulty in the investigation of cell migration is in relevant techniques. In vivo observation of live-cell migration (not long distance migration, yet) by means of the time-lapse live-cell imaging technique has been started only very recently for some simplest systems, such as a cluster of border cells in a Drosophila ovary [8]. However, there is still a big step between this experiment and observation of live-cell migration in meridians in vivo, and that is why investigations on cell migration in 3D ECM have been carried out mostly with theoretical models [8], instead of experiments, despite their obvious importance.

The mysterious meridian function discovered by Chinese medicine is still only a mystery rather than science, but it is an important mystery that should not be neglected any longer by real sciences like physiology. Now, cell migration and mast cell function, the two major blocks to disclosure of the meridian function mystery, have already become hot topics and people from different fields like molecule biology, cell biology, histology, physiology, and medical physiology are making all their efforts to tackle the two down, so we believe that, despite the fact that many difficulties will be encountered, the day when the meridian function mystery transforms into science is not far anymore.

## 7. Summary

Firstly, based on the latest knowledge on cell migration and starting from the statement that meridians are zones in the loose connective tissue containing relatively richer interstitial fluid [4], we argue that meridians are lower-resistance passages for cell migration.

Secondly, considering that meridians are lower-resistance pathways for both cell migration and diffusion of cell mediators (such as histamine [5]), we further argue that meridians are lower-resistance passages for chemotactic migration of cells.

Then, according to some details in the mechanism of cell migration that have been disclosed recently, we point out that the results of an earlier histological study on mast cells in subcutaneous connective tissue (fascia) in albino rats [16] already indicate that for the adult rats mast cells are often in chemotactic migration in meridians longitudinally; that is, mast cells may make longitudinal chemotactic migration along meridians.

Finally, we point out that by adding the statement that “meridians are lower-resistance passages for chemotactic migration of cells” and “mast cells may make longitudinal chemotactic migration along meridians” to the hypothesis for the meridian function [5], which suggests that in addition to the loose connective tissue network the circulatory and nervous systems are also involved and functioning coordinately for the meridian function, and recalling that mast cells have been known recently to be versatile regulators of inflammation, tissue remodeling, host defense, and homeostasis [18], one may now start to think of or even to do some evidence-based experiments to reveal the real scientific meaning behind the so-called “*circulating “air and blood”,*” “*transferring responses,*” “*reflecting symptoms,*” and “*resisting diseases,*” that is, the core of the meridian doctrine.

## Figures and Tables

**Table 1:** Comparison of the two migration strategies of single cells [6, 9].

Migration type	Mesenchymal	Amoeboid

Migration strategy	Path generating	Path finding

Mechanism for overcoming tissue barriers	Proteolytic matrix defect	Morphological adaption (constriction ring)

Composition of cell-matrix interactions	Focalized; integrins and MT1-MMP coclustered	Diffuse; integrins nonclustered; MT1-MMP internalized and dissociated from integrins

Migration speed	Very low (formation and turnover of focal contacts are time-consuming; in 3D models it is only 0.1–2 *μ*/min)	Very high (for resting and activated leukocytes it may reach 30 *μ*/min in vivo)

**Table 2:** Major mast-cell-derived mediators and their physiological effects [22].

Class	Mediators	Physiological effects
Performed mediators	Histamine, serotonin, heparin, neutral proteases (tryptase and chymase, carboxypeptidase, cathepsin G), major basic protein, hydrolase, peroxidase, phospholipases	Vasodilation, vasoconstriction, angiogenesis, mitogenesis, pain, protein processing/degradation, lipid/proteoglycan, arachidonic acid generation, tissue damage, inflammation

Lipid mediators	LTB_4_, LTC_4_, PGE_2_, PGD_2_, PAF	Leucocyte chemotaxis, vasoconstriction, bronchoconstriction, platelet activation, vasodilation

Cytokines	TNF-*α*, TGF-*β*, IFN-*α*, IFN-*β*, IFN-*γ*, IL-1*α*, IL-1*β*, IL-3, IL-4, IL-5, IL-6, IL-8, IL-9, IL-10, IL-11, IL-12, IL-13, IL-15, IL-16, IL-18, IL-25, SCF, MIF	Inflammation, leucocyte migration/proliferation

Chemokines	CXCL8, CCL3, CCL2, CCL7, CCL13, CCL5, CCL11, CCL19	Chemoattraction and tissue infiltration of leucocytes

Growth factors	CSE, GM-CSF, bFGF, VEGE, NGF, LIF	Growth of various cell types, vasodilation, neovascularization, angiogenesis

SCF, stem cell factor; GM-CSF, granulocyte macrophage-colony stimulating factor; VEGF, vascular endothelial growth factor; bFGF, basic fibroblast growth factor; NGF, nerve growth factor.
